# Pulmonary Type B Niemann-Pick Disease Successfully Treated with Lung Transplantation

**DOI:** 10.1155/2019/9431751

**Published:** 2019-06-16

**Authors:** R. S. O'Neill, N. Belousova, M. A. Malouf

**Affiliations:** St Vincent's Hospital Department of Lung Transplant, Sydney, NSW, Australia

## Abstract

**Background:**

Niemann-Pick Disease (NPD) type B is a rare autosomal recessive disease characterised by hepatosplenomegaly and pulmonary disease, highlighted by preserved volumes and diminished diffusion capacity of the lung for carbon monoxide (DLCO) on pulmonary function tests (PFTs). There is no current accepted treatment for the disease. We present a case of a successful bilateral lung transplant in a patient with a DLCO of 14%, and significant pulmonary changes attributable to NPD type B on computed tomography (CT) chest, and both microscopic and macroscopic assessment of the lung explant. To the author's knowledge this is only the third case of lung transplantation in a patient with NPD type B and is one of two current living patients post lung transplantation for NPD type B.

**Case Report:**

A 64-year-old male patient underwent bilateral lung transplantation for NPD type B. Preoperative PFTs demonstrated preserved volumes with significantly decreased DLCO, with imaging showing a diffuse reticular interstitial pattern, typical of chronic fibrotic lung disease. The patient suffered from primary graft dysfunction type 3 in the postoperative period as well as rejection managed with methylprednisolone and intravenous immunoglobulin. The patient improved steadily and was discharged 80 days post-transplantation.

**Conclusions:**

This case is only the third reported case of lung transplantation in a patient with NPD type B and the second case of a patient with NPD type B currently living post-transplantation, being at postoperative day (POD) 267 at the time of manuscript drafting. It demonstrates that lung transplantation, although hazardous, is a viable strategy for treatment in patients with NPD type B who have significant pulmonary involvement.

## 1. Background

Niemann-Pick disease (NPD) is a member of the family of lysosomal storage disorders and refers to patients who present with lipid storage and tissue foam cell infiltration, along with clinical features such as hepatosplenomegaly, pulmonary insufficiency, and potentially central nervous system (CNS) involvement [1]. The incidence of the disease is not well documented and current estimates are thought to be inaccurate due to previous studies identifying patients through clinician directed biochemical testing for enzymatic confirmation, rather than established population-based screening programs [2]. There has been minimal research into the ethnic predominance of NPD; however studies in the Ashkenazi Jewish population have identified a carrier frequency of approximately 1:90 for the gene mutations causing NPD type A, with an estimated birth rate of approximately 3 per 100,000 [3].

The disease is subclassified into three main types; A, B, and C, with types A and B being associated with the sphingomyelin phosphodiesterase 1 gene (SMPD_1_) mutation resulting in a deficiency in acid sphingomyelinase (ASM) activity. Currently over 180 mutations in the SMPD_1_ gene have been identified, resulting in NPD type A or B phenotypes [4]. NPD type C is genetically distinct and separate from types A and B and is attributed to mutations of either the NPC1 or NPC2 genes [5].

The characteristic clinical syndrome of NPD type B is defined by recurrent respiratory infections, interstitial lung disease, hypoxia, hepatosplenomegaly, liver cirrhosis, and portal hypertension, with the age of clinical presentation ranging from early childhood to the fourth or fifth decade of life [6, 7]. Neurological manifestations are less frequent but can also be present.

Diagnosis requires the demonstration of reduced ASM activity in isolated leukocytes or cultured skin [8]. A recent review highlighted the pulmonary changes associated with NPD type B include preservation of normal lung volume and decreased diffusion capacity of carbon monoxide (DLCO) on pulmonary function tests (PFTs). A reticular or reticulonodular pattern is present on chest radiography, with ground glass opacities, thickening of the interlobular septa and intralobular lines, and a lower lobe predominance identified on computed tomography (CT) [6, 9, 10].

The majority of mortality associated with NPD type B occurs in the paediatric population, with a recent study reporting a mortality rate of 19% and a median age of death of 15.5 years. Their results suggested that disease severity in childhood predicted survival, with fulminant hepatic failure, pneumonia, and complications of bone and stem cell transplants being cited as causes of mortality in this cohort [11]. In addition to this, splenectomy is another factor that has been associated with an increase in mortality, with both this and the early onset of disease being indicators of disease severity [12].

There is no current accepted treatment for NPD type B with supportive care being the mainstay of therapy; however clinical trials have recently focused on enzyme replacement therapy as a mode of treatment [13]. Lung transplantation for those with significant pulmonary pathology is in its infancy with only two cases being published in October 2018 and January 2019, with one dying 29 days post-transplant and the other reported to be alive at 35 months post-transplant [14, 15].

The present article describes successful lung transplantation in a patient with significant interstitial lung disease and pulmonary hypertension attributable to NPD type B.

## 2. Case Report

A 64-year-old Maltese male with NPD type B diagnosed on genetic studies 29 years prior after splenectomy for a splenic rupture was evaluated for lung transplantation. He had a significant medical history, diagnosed with both pulmonary and portal hypertension attributable to NPD type B. The patient had a 70-pack year smoking history (ceased in 2010) with no family history of NPD.

The patient was evaluated for lung transplantation due to functional impairment, characterised by a 24-hour oxygen requirement, a baseline oxygen saturation of 73% on 6 litres of oxygen, and a significant exercise limitation, with a six-minute walk test result being 50% of predicted and a post-test oxygen saturation of 65%. His pre-transplant PFTs demonstrated preserved lung volumes and a significantly reduced adjusted DLCO of 14%. Increased pulmonary vascular resistance was found on cardiac catheterisation, with a mean pulmonary pressure of 41 mmHg. Right ventricular dilatation was identified on pre-transplant echocardiogram with mild biatrial dilatation; however systolic function was normal. Liver function tests pre-transplant were normal aside from an elevated total bilirubin, 57 *μ*mol/L, 11 *μ*mol/L conjugated and 46 *μ*mol/L unconjugated.

**Figure 1:**
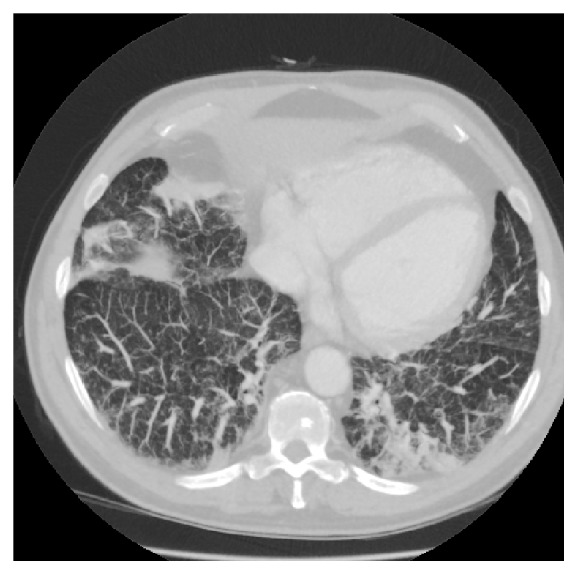
Chest computed tomography of the patient's native lungs demonstrating a diffuse reticular, fibrotic, interstitial pattern.

Further to this, pre-transplantation CT demonstrated a diffuse reticular interstitial pattern of typical chronic fibrotic lung disease, worse in the subpleural zones and at the bases, with subpleural blebs in the left lower zone, consistent with pulmonary NPD type B. A small triangular density was present in the left posterior side, deemed to be a focal consolidation (Figure 1). Pre-transplant serology demonstrated previous Cytomegalovirus and Epstein-Barr virus infection in the recipient.

The patient received a bilateral lung transplant from a donor positive for hepatitis B virus infection which was treated with preoperative entecavir. The patient was on cardiopulmonary bypass intraoperatively for 223 minutes with an intraoperative airway reperfusion injury managed with 60 mg of intravenous furosemide and intraoperative hypotension requiring pharmacological vasopressor support both intraoperatively and postoperatively. The cold ischaemic time was 295 and 205 minutes for the right and left lung respectively.

The patient was commenced on cefotaxime pre-transplant as per institutional protocol and was subsequently changed to flucloxacillin due to donor swabs and day 1 bronchoalveolar lavage (BAL) growing methicillin sensitive* Staphylococcus aureus *(MSSA). On day 2 post-transplant, ceftazidime was initiated due to several episodes of pyrexia. This was escalated to cefazolin and meropenem on day 6 post-transplant due to an acute liver injury and ongoing hypotension. Vancomycin and gentamicin were also initiated under the presumption that the patient was septic; however blood cultures were negative during admission. Cefazolin was ceased after 24 hours and meropenem was continued until day 21 post-transplant. In addition to antibiotic coverage, anidulafungin was initiated on day 6 post-transplant for antifungal cover in the context of sepsis. This was used in replacement of voriconazole due to the acute liver injury identified in the intensive care unit (ICU).

Postoperative histological analysis of the explanted native lungs demonstrated features of lipoid pneumonia, in keeping with pulmonary NPD. Post-transplantation induction immunosuppression was initiated with basiliximab, followed by tacrolimus, mycophenolate, and prednisone.

The postoperative period was complicated by primary graft dysfunction type 3 and a vasoplegic state requiring vasopressor support in the immediate and ongoing postoperative period. The patient developed an anuric acute kidney injury (AKI) requiring haemodialysis; and paroxysmal atrial fibrillation (pAF) with haemodynamic instability requiring treatment with direct current (DC) cardioversion, an amiodarone infusion, and digoxin. Chest radiograph in the ICU demonstrated pneumomediastinum and bilateral pleural effusions. Vancomycin resistant enterococcus (VRE) was cultured from the pleural fluid and subsequently managed with intravenous linezolid. Other complications included recurrent rhinovirus infection, bilateral cephalic vein thrombi; an upper gastrointestinal haemorrhage managed with transfusion, endoscopic haemostasis, and angioembolization; and cerebral ischaemic changes highlighted by a frontal lobe infarction noted on CT brain.

**Figure 2:**
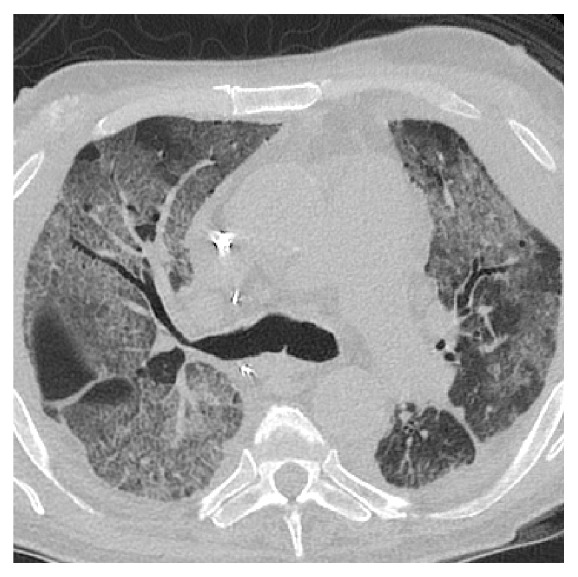
Transverse chest computed tomography demonstrating widespread peribronchovascular ground-glass opacities throughout both lung fields.

On day 45 post-transplantation, the patient was diagnosed with clinical acute cellular rejection (ACR) and antibody mediated rejection (AMR) characterised by evidence of donor specific antibodies to HLA DQ7 and DGA1*∗*05:05 in the recipient serum. High resolution computed tomography (HRCT) chest demonstrated widespread peribronchovascular ground-glass opacities throughout both lungs (Figure 2). This was managed with three doses of methylprednisolone and intravenous immunoglobulin. The patient was discharged after 80 days with repeat bronchoscopy revealing no evidence of anastomotic breakdown. Bronchial wash at the time grew* Pseudomonas aeruginosa *sensitive to ciprofloxacin and tazocin. The patient was no longer limited by breathlessness and the remaining sequalae of his AKI resolved in November 2018, with haemodialysis no longer required.

To date the patient has required two further hospitalisations, one for profound hypoxia and respiratory sepsis attributed to* Pseudomonas aeruginosa. *Imaging at the time demonstrated widespread ground glass changes. The most recent admission, for hypoxia, was attributed to pulmonary oedema and a concurrent lower respiratory tract infection.

## 3. Discussion

Since the first successful lung transplant in 1963, there has been a steady increase in the need for lung transplantation worldwide [16]. Australia has improved its donor rates from being one of the lowest per year to 18 donors per million of the population [17]. This is due in part to the increased rates of donation from deaths which occur after circulatory death (DCD) and Ex Vivo Lung Perfusion (EVLP) increasing the donation rate by 25% overall. Despite this, lung transplantation is still limited due to a shortage of organ donors [18]. The aim of lung transplantation is the alleviation of respiratory symptoms with improvement in both survival and quality of life. The survival rate of patients post-lung transplant is approximately 50% at 5 years with the 10-year survival rate decreasing to 20%, which is low compared to other solid organ transplants [19]. This is due to the development of chronic lung allograft dysfunction affecting the long-term survival post-transplant [20].

NPD is a rare autosomal recessive disorder characterised by a deficiency in activity of the enzyme ASM. Patients can present at any time, with NPD type B having a variable disease course associated with a broad spectrum of disease severity and clinical manifestations. The most common clinical manifestation is hepatosplenomegaly which is identified during early childhood [21, 22]. Other clinical features include diminished pulmonary function, specifically an impaired DLCO with preserved volumes, myelosuppression, and neurological features in variant forms of the disease [7].

The current literature on therapeutic interventions for NPD type B is limited with there being no consensus on the treatment of ASM deficiency. Haematopoietic stem cell transplantation (HSCT) has been demonstrated to reduce liver and spleen size, as well as treat pulmonary involvement, however the complications associated with this method are well documented [23, 24]. In addition to HSCT, whole lung lavage has also been shown to have some therapeutic efficacy [25, 26].

This is only the third documented lung transplant in a patient with NPD type B with pulmonary involvement. The two previous recorded cases produced variable results, one patient only living to 29 days post-transplant with autopsy demonstrating NPD like changes in the donor lungs, suggesting disease recurrence, and the second still reported to be living at 35 months post-transplant [14, 15].

## 4. Conclusion

This case is only the third documented case of lung transplantation in a patient with NPD type B. It is one of two recorded cases in the literature of a current living patient with NPD type B post-lung transplantation with the only other living case being reported to be 35 months post-transplant at the time of manuscript drafting. The only unsuccessful case died 29 days post-transplant as a result of shock, multiorgan failure and autopsy demonstrating pulmonary NPD involvement in the donor lungs [14, 15]. Although the peri- and postoperative period in the presented patient was not without its complications, it does present a potentially viable treatment option in patients whom are medically suitable for transplantation with clinically significant pulmonary disease attributable to a systemic metabolic disease.
